# Supplementary material for: Interface engineering of Ta3N5 thin film photoanode for highly efficient photoelectrochemical water splitting
Source: Nat Commun. 2022 Feb 7;13:729. doi: 10.1038/s41467-022-28415-4 (PMC8821563; doi:10.1038/s41467-022-28415-4)
Supplement: Supplementary file 1 — Supplementary Information [file 41467_2022_28415_MOESM1_ESM.pdf]

Supplementary Information

Interface engineering of Ta_3N_5 thin film photoanode for highly efficient photoelectrochemical water splitting

Jie Fu,[1] Zeyu Fan,[1,2] Mamiko Nakabayashi,[3] Huanxin Ju,[4] Nadiia Pastukhova,[1] Yequan Xiao,[1] Chao Feng,[1] Naoya Shibata,[3] Kazunari Domen,[5,6] & Yanbo Li[1, 2,*]

[1]Institute of Fundamental and Frontier Sciences, University of Electronic Science and Technology of China, Chengdu 610054, China.

[2]Yangtza Delta Region Institute (Huzhou), University of Electronic Science and Technology of China, Huzhou 313001, China.

[3]Institute of Engineering Innovation, The University of Tokyo, Tokyo 113-8656, Japan.

[4]PHI China Analytical Laboratory, CoreTech Integrated Limited, Nanjing 211111, China.

[5]Office of University Professors, The University of Tokyo, Tokyo 113-8656, Japan

[6]Research Initiative for Supra-Materials (RISM), Shinshu University, Nagano 380-8553, Japan

[*]E-mail: yanboli@uestc.edu.cn

Supplementary Note 1:

Stretched-exponential decay fitting. As shown in Supplementary Fig. 8, the TRPL decays were fitted by using a stretched-exponential decay model:

$$I(\mathrm{t}) = \int_{-\infty}^{t} IRF(t') \sum_{i=1}^{n} A_i e^{-(\frac{t-t'}{\tau_i})^{\beta}} \mathrm{d}t' \quad (1)$$

where A_i, τ, and β stand for the initial luminescence intensity following the excitation, the decay time for photogenerated carriers, and the trap-related stretching component, respectively. IRF is the measured instrument response function of the TRPL system, which is used as the reference to fit the decay with a numerical re-convolution algorithm. The detailed fitting parameters are listed in Supplementary Table 1. The average lifetime of the stretched exponential decay ⟨τ⟩ is calculated following the equation[1, 2]:

$$<\tau> = \frac{\tau}{\beta} \Gamma(\frac{1}{\beta}) \quad (2)$$

where $\Gamma(\frac{1}{\beta})$ is defined as the gamma function[3]:

$$\Gamma(\frac{1}{\beta}) = \int_{0}^{\infty} x^{(1-\beta)/\beta} e^{-x} dx \quad (3)$$

Supplementary Note 2:

Surface charge injection efficiency (η_{inj}) and bulk charge separation efficiency (η_{bulk}). The photocurrent density (J_{H_2O}) obtained during the PEC water oxidation measurement could be represented as:

$$J_{H_2O} = J_{abs} \times \eta_{bulk} \times \eta_{inj} \quad (4)$$

where J_{abs} is the maximum photocurrent density generated by assuming all the absorbed photons in the sample are converted into photocurrent:

$$J_{abs} = q\int \Phi_\lambda [1 - \exp(-\alpha\lambda d)] d \quad (5)$$

where q, λ, Φ_λ, d, and α stand for the charge of single electron, photon wavelength, photon flux of standard solar spectrum (AM 1.5G), film thickness, and absorption coefficient of the film, respectively.

When H_2O_2 is added as a scavenger into 1 M KOH electrolyte, it is considered there is no injection barrier of holes for H_2O_2 oxidation. Therefore, the surface charge injection efficiency (η_{inj}) is calculated by:

$$\eta_{inj} = (J_{H_2O} / J_{H_2O_2}) \times 100\% \quad (6)$$

The bulk charge separation efficiency (η_{bulk}) is calculated by dividing the photocurrent density achieved for H_2O_2 oxidation ($J_{H_2O_2}$) by J_{abs}:

$$\eta_{bulk} = (J_{H_2O_2} / J_{Abs}) \times 100\% \quad (7)$$

Supplementary Note 3:

Open circuit potential (OCP) decay derived carrier lifetime. The OCP decay profiles of Ta_3N_5-based photoanodes with different layered structures were terminated after illumination for 10 min to create significant charge recombination. To compare photogenerated charge lifetime and recombination rate at photoanode/electrolyte interface, the carrier lifetime was calculated by:

$$\tau_n = -\frac{k_B T}{e}\left(\frac{\mathrm{dOCP}}{\mathrm{dt}}\right)^{-1} \tag{8}$$

where τ_n, k_B, T, and e stand for the potential-dependent carrier lifetime, the Boltzmann's constant, temperature in Kelvin, and the charge of single electron, respectively.

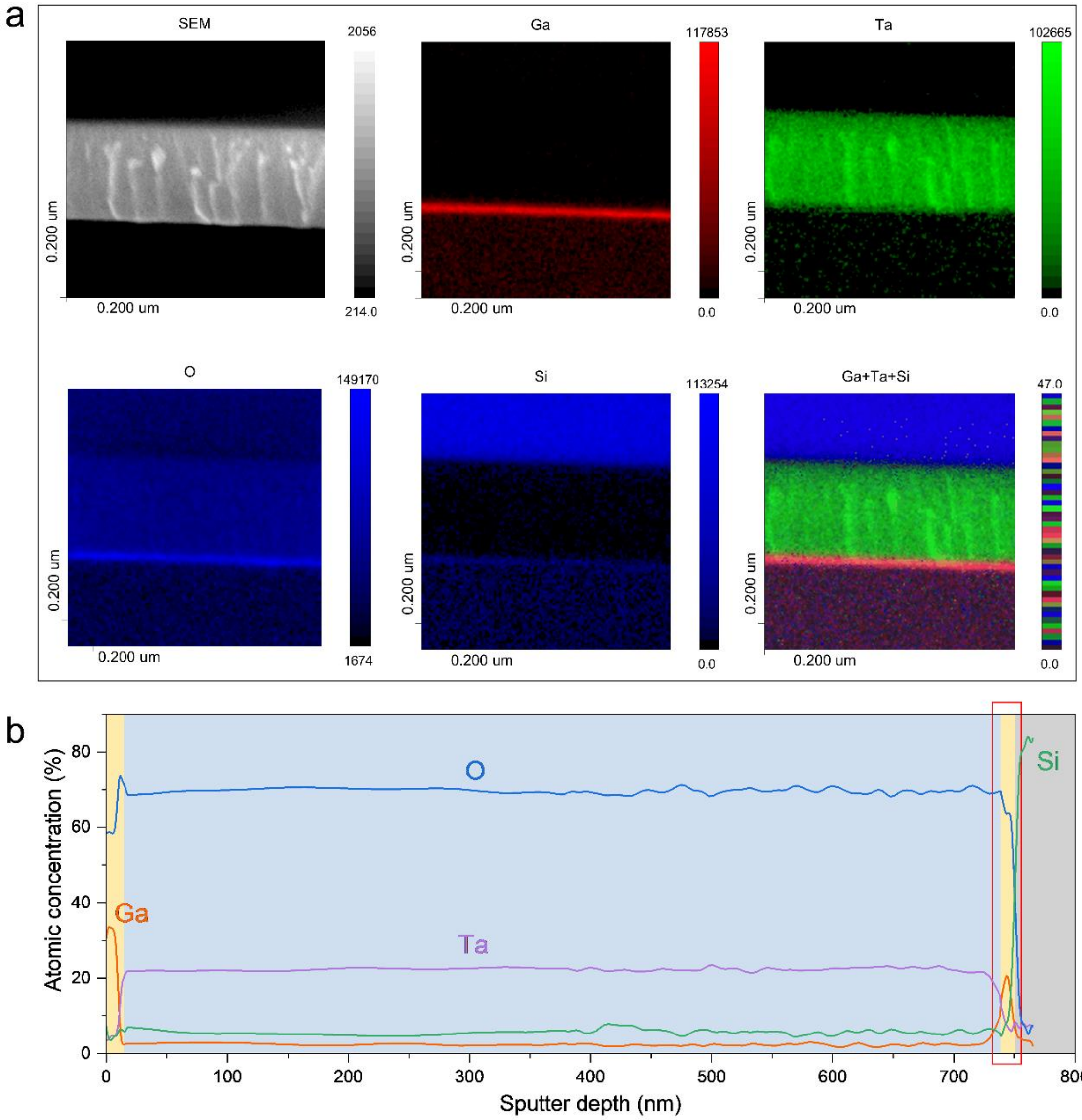

Supplementary Fig. 1 | Structural characterizations of the InO_x-GaO_x/TaO_x/ Mg:GaO_x precursor film deposited on Si substrate. a, Cross-sectional SEM image and AES elemental mapping of the film. Note that the film is facing down in the images. Si substrate was used in order to make a clear cleavage of the film. **b**, AES depth profile of the multilayer oxide precursor film. The GaO_x layer on top of the TaO_x layer was clearly observed by AES mapping, while the GaO_x layer at the Si/TaO_x interface was not directly observed due to its thin thickness. However, the AES depth profile clearly revealed that there was a Ga-containing layer at the Si/TaO_x interface, as highlighted in the red square. For In and Mg dopants, their concentrations were probably below the detection limit of the AES instrument.

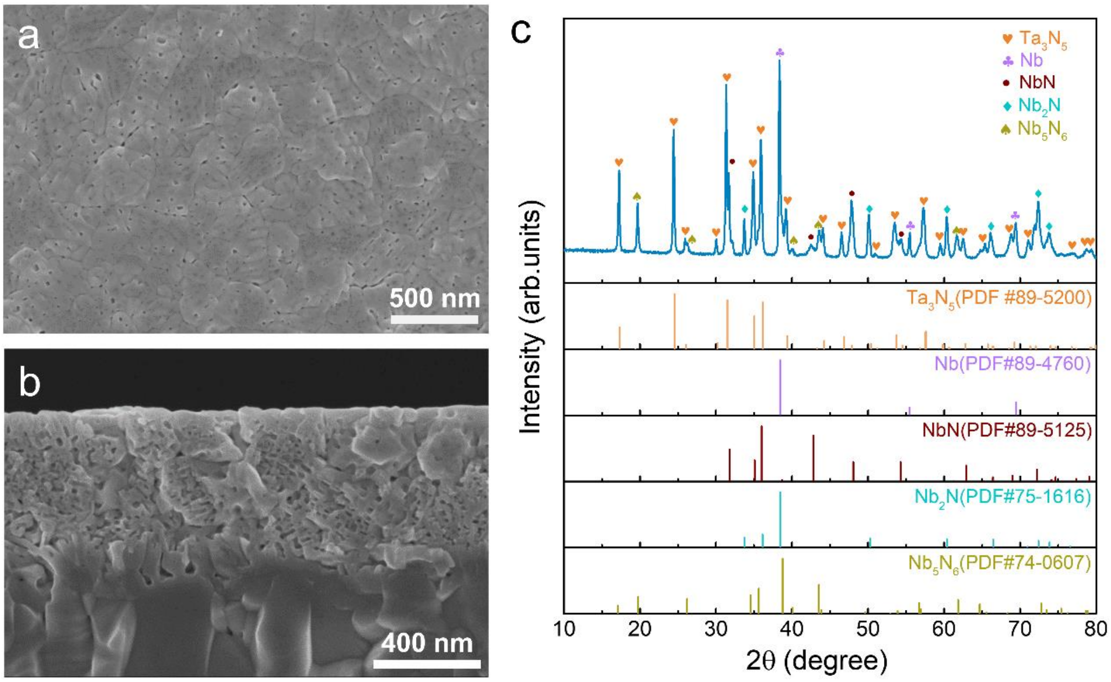

Supplementary Fig. 2 | The morphology and structure of In:GaN/Ta_3N_5/Mg:GaN thin film deposited on Nb substrate. a, Top-view and **b,** cross-sectional SEM images of In:GaN/Ta_3N_5/Mg:GaN thin film. **c**, XRD pattern of In:GaN/Ta_3N_5/Mg:GaN thin film on Nb substrate.

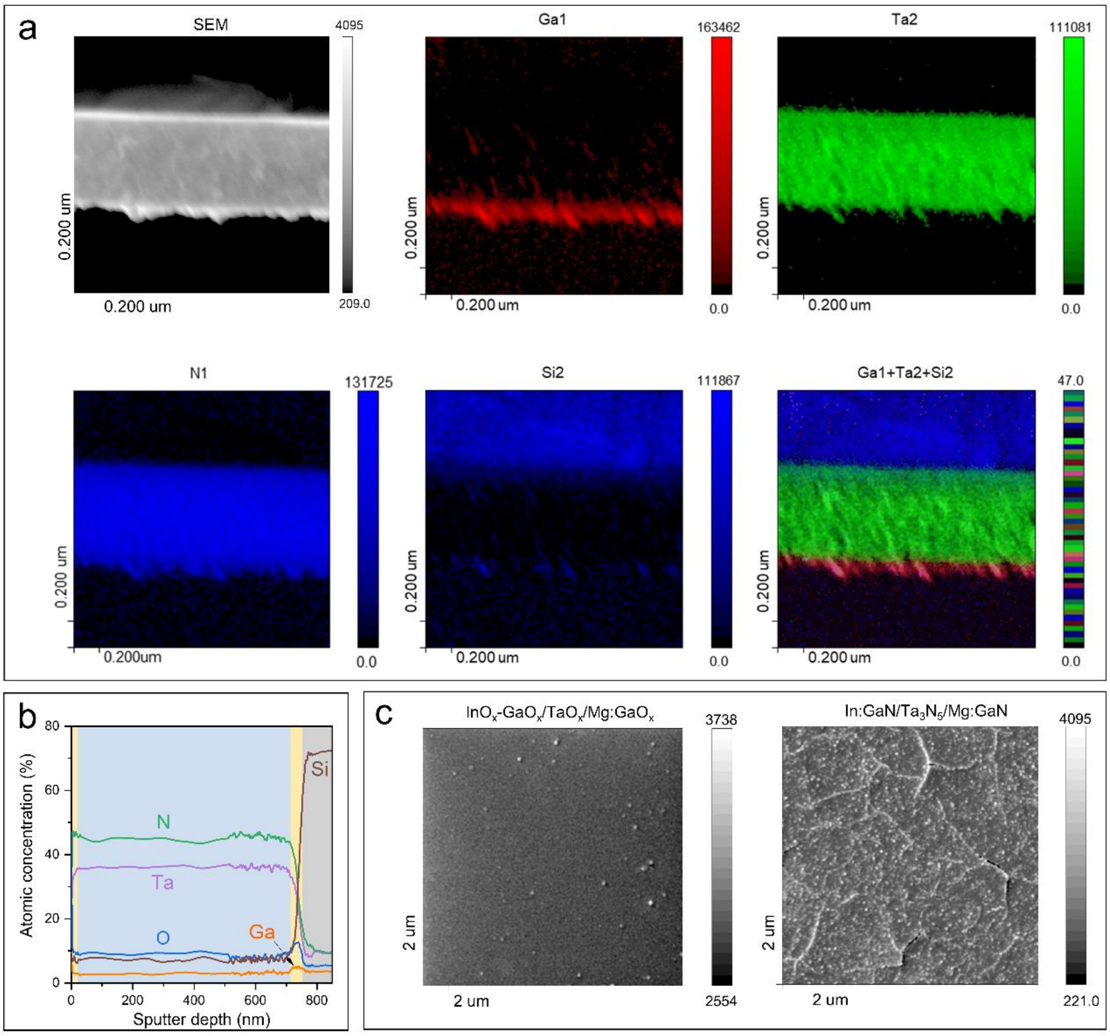

Supplementary Fig. 3 | Structural characterizations of the In:GaN/Ta_3N_5/ Mg:GaN film deposited on Si substrate. a, Cross-sectional SEM image and AES elemental mapping of the film. **b**, AES depth profile of the Si/In:GaN/Ta_3N_5/Mg:GaN film. **c**, Top-view SEM images of the InO_x-GaO_x/TaO_x/ Mg:GaO_x precursor film and the In:GaN/Ta_3N_5/Mg:GaN film. Due to the increased surface roughness after nitridation, the resolution of the AES depth profile for the In:GaN/Ta_3N_5/Mg:GaN film was not as good as that for the InO_x-GaO_x/TaO_x/Mg:GaO_x film. Nevertheless, it is still able to reveal a Ga-containing interlayer at the Si/Ta_3N_5 interface.

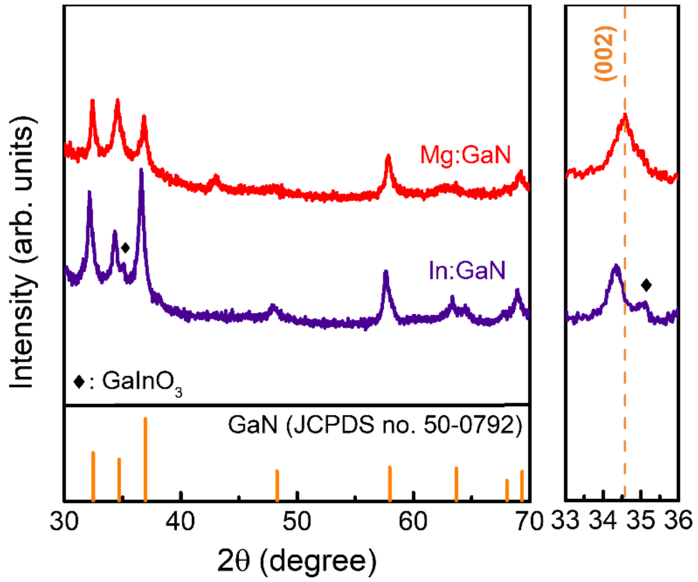

Supplementary Fig. 4 | XRD patterns of In:GaN and Mg:GaN thin film deposited on quartz glass substrates.

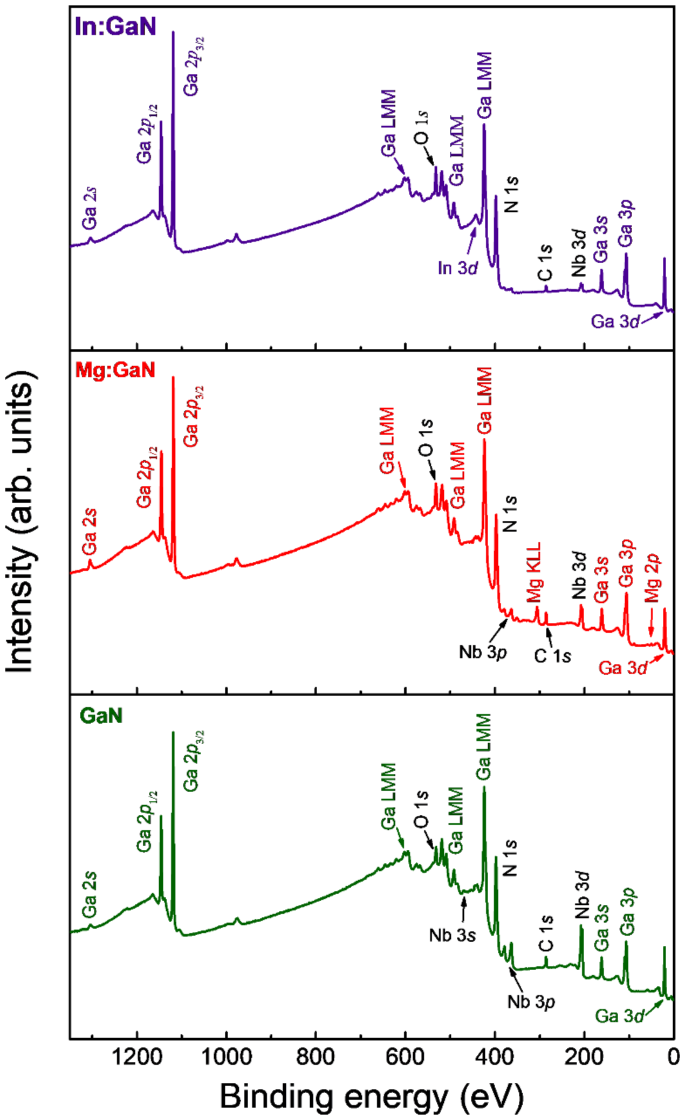

Supplementary Fig. 5 | The XPS survey spectra of GaN, Mg:GaN and In:GaN films deposited on Nb substrate. Charging shift compensation was made for all curves using C 1s at 284.8 eV as the reference.

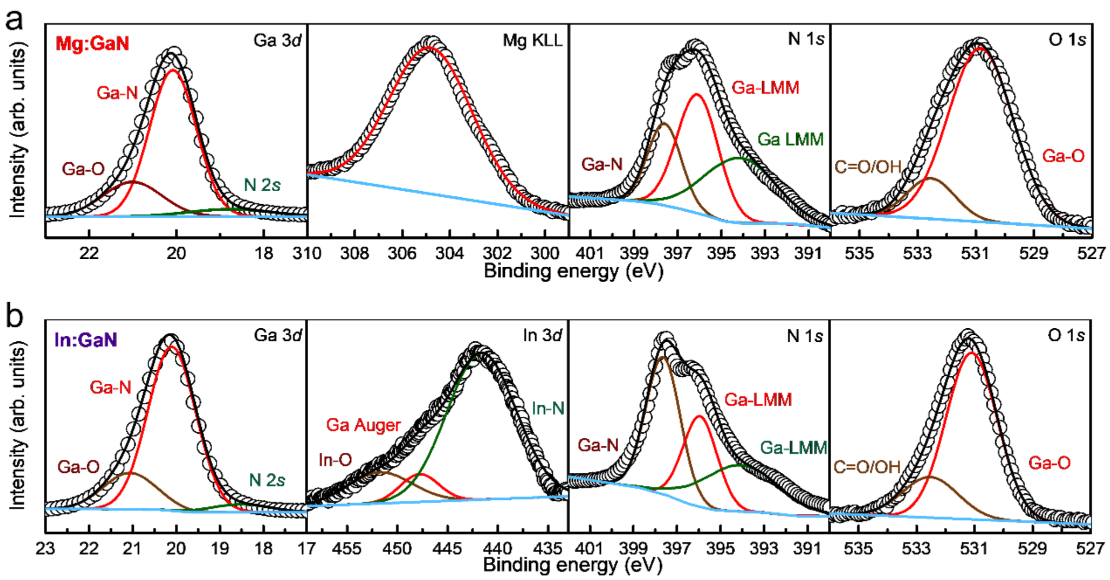

Supplementary Fig. 6 | The XPS core-level spectra of **a,** Mg:GaN and **b,** In:GaN films deposited on Nb substrate.

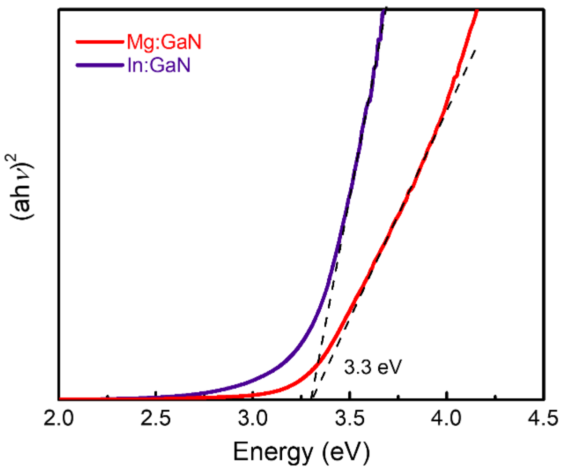

Supplementary Fig. 7 | Tauc plots of UV-vis absorption spectra for In:GaN and Mg:GaN films deposited on quartz glass substrates.

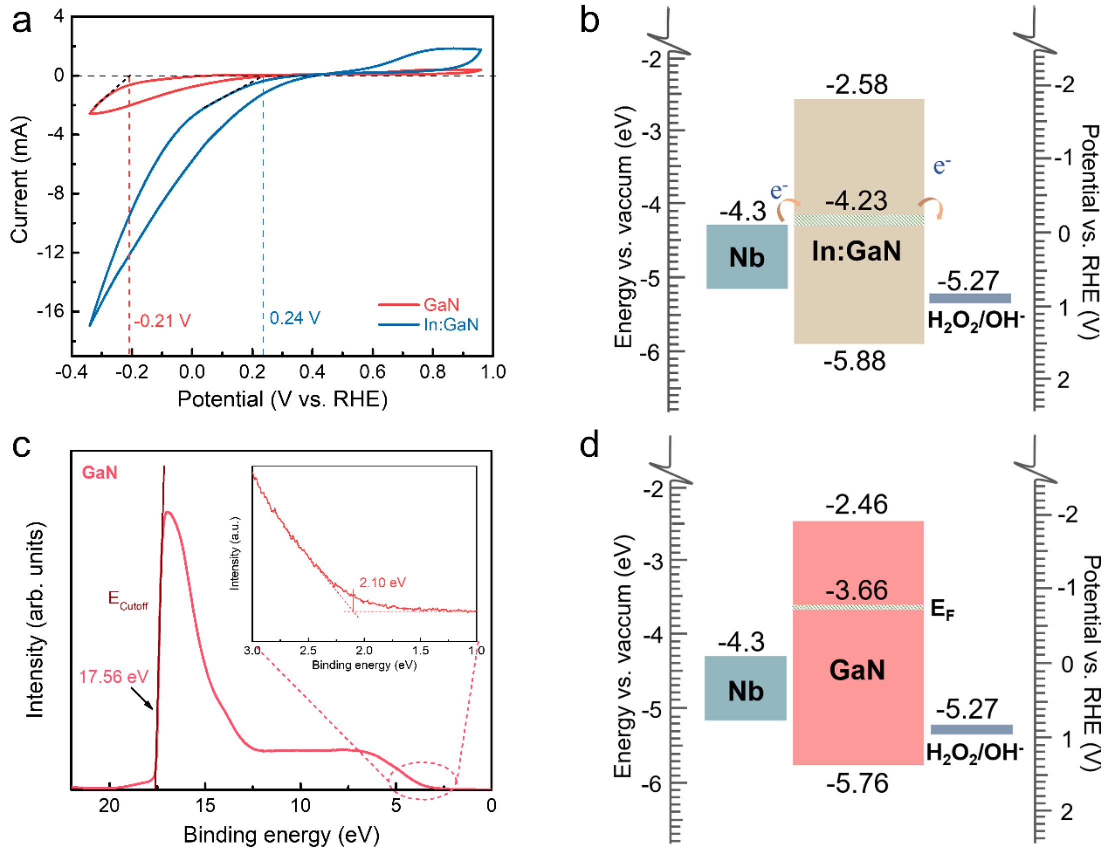

Supplementary Fig. 8 | Electron transport properties of In:GaN and GaN films. The In:GaN and GaN compact films were obtained by nitriding In-doped GaO_x and pure GaO_x films deposited on Nb substrates through dual-source electron beam evaporation. **a**, Cyclic voltammetry of compact In:GaN and GaN films measured in 1 M KOH with 0.5 M H_2O_2 as a scavenger. **b**, Schematic energy diagram showing there is almost no barrier for the injection of electrons from the Nb electrode through the In-induced inter-gap state to the electrolyte. **c**, UPS spectrum of GaN film deposited on Nb substrate. **d**, Schematic energy diagram showing there is a relatively high barrier for the injection of electrons from the Nb electrode through the GaN layer to the electrolyte. These results verified that the In-induced inter-gap state can indeed act as a channel for electron transport through the In:GaN layer, resulting in the more positive onset potential and higher current value for the reduction current observed in **a**.

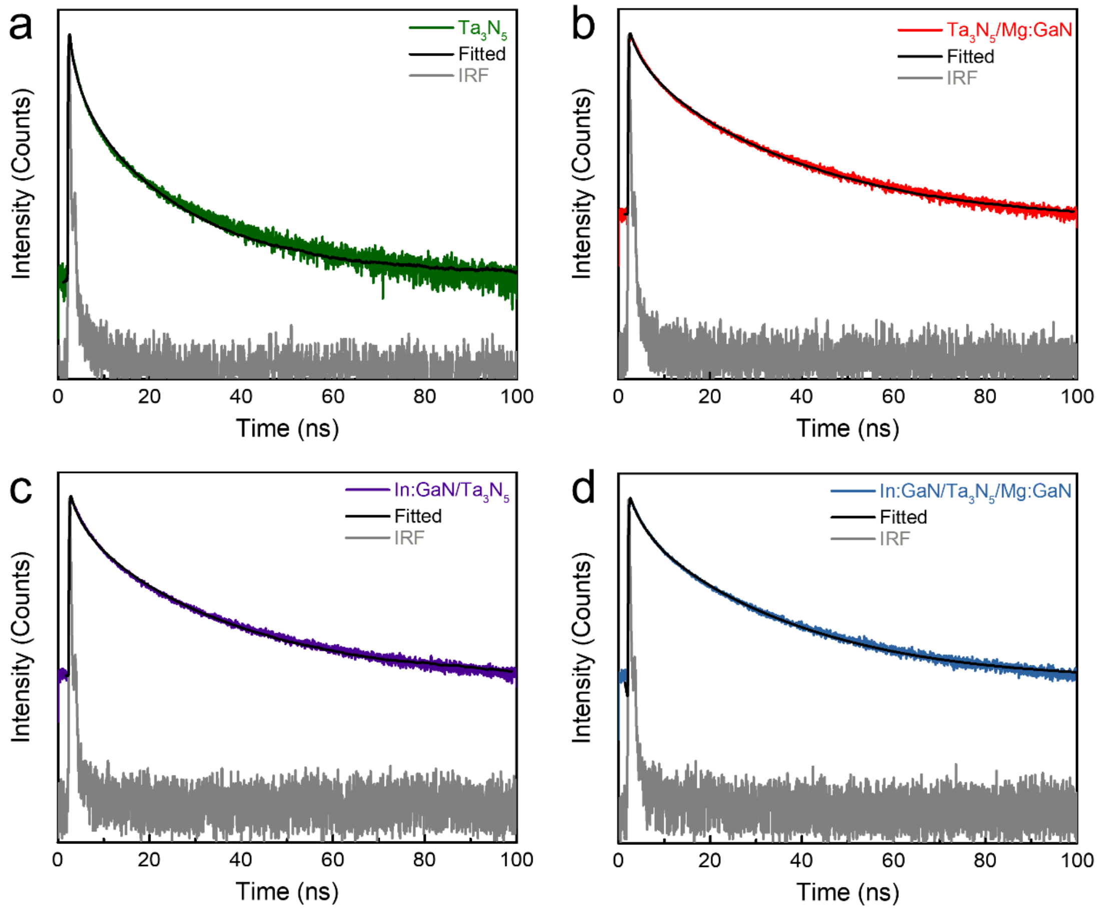

Supplementary Fig. 9 | The TRPL decay curves of four Ta_3N_5-based films with different layered structures on quartz glass substrate. a, Ta_3N_5. **b,** Ta_3N_5/Mg:GaN. **c,** In:GaN/Ta_3N_5. **d,** In:GaN/Ta_3N_5/Mg:GaN. The TRPL curves were acquired at 475 nm with 20 nm bandwidth under the excitation of a 375-nm picosecond laser pulsed at a repetition rate of 1 MHz. The system instrument response function (IRF) was measured and used as the reference to fit the decay with a numerical re-convolution algorithm. All TRPL decay curves can be well-fitted using a stretched exponential decay model.

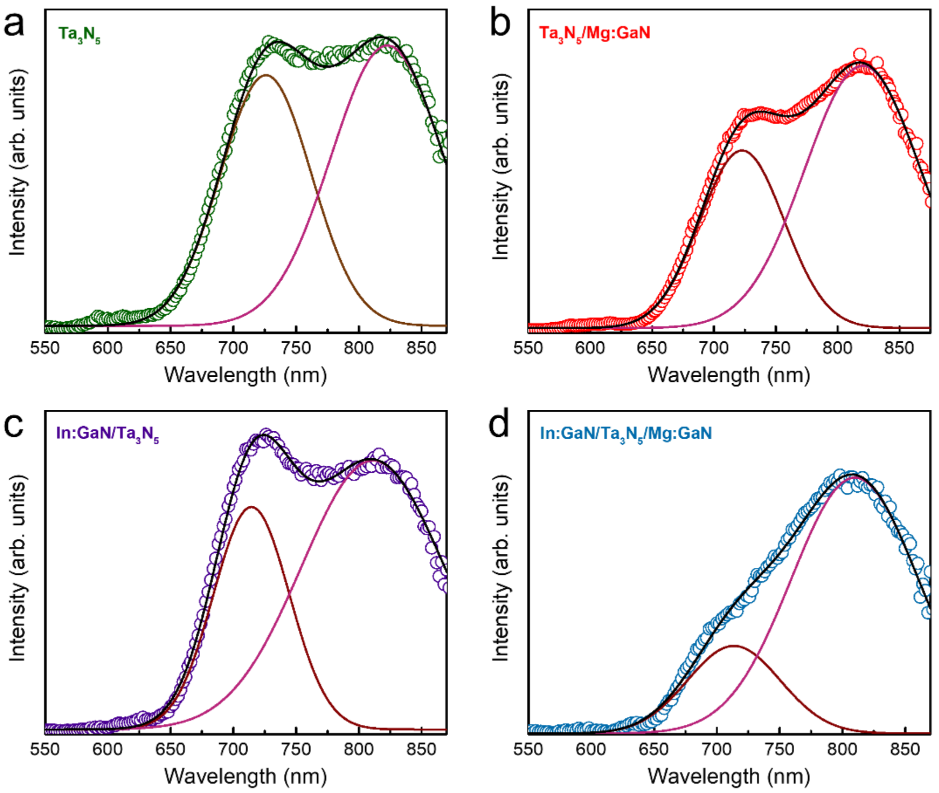

Supplementary Fig. 10 | Deconvolution of PL spectra of four different structures Ta_3N_5-based thin films measured under 510 nm pulsed laser excitation at 8 K. a, Ta_3N_5. **b,** Ta_3N_5/Mg:GaN. **c,** In:GaN/Ta_3N_5. **d,** In:GaN/Ta_3N_5/Mg:GaN. All the spectra can be well-deconvoluted into two peaks centered at ~720 nm (1.72 eV) and ~820 nm (1.51 eV), respectively.

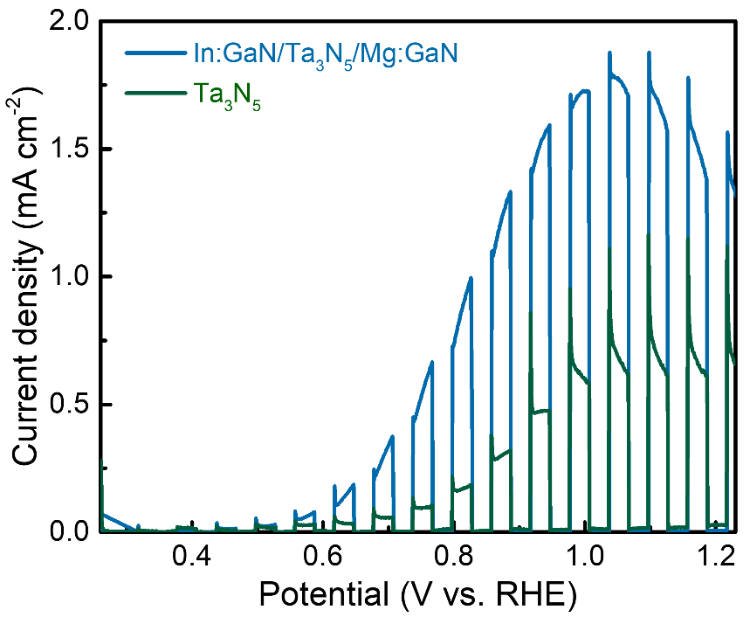

Supplementary Fig. 11 | PEC performance of the Ta_3N_5 and In:GaN/Ta_3N_5/Mg:GaN photoanodes on Nb substrate without co-catalyst modification. The J-V curves were measured under chopped AM 1.5G illumination in 1 M KOH.

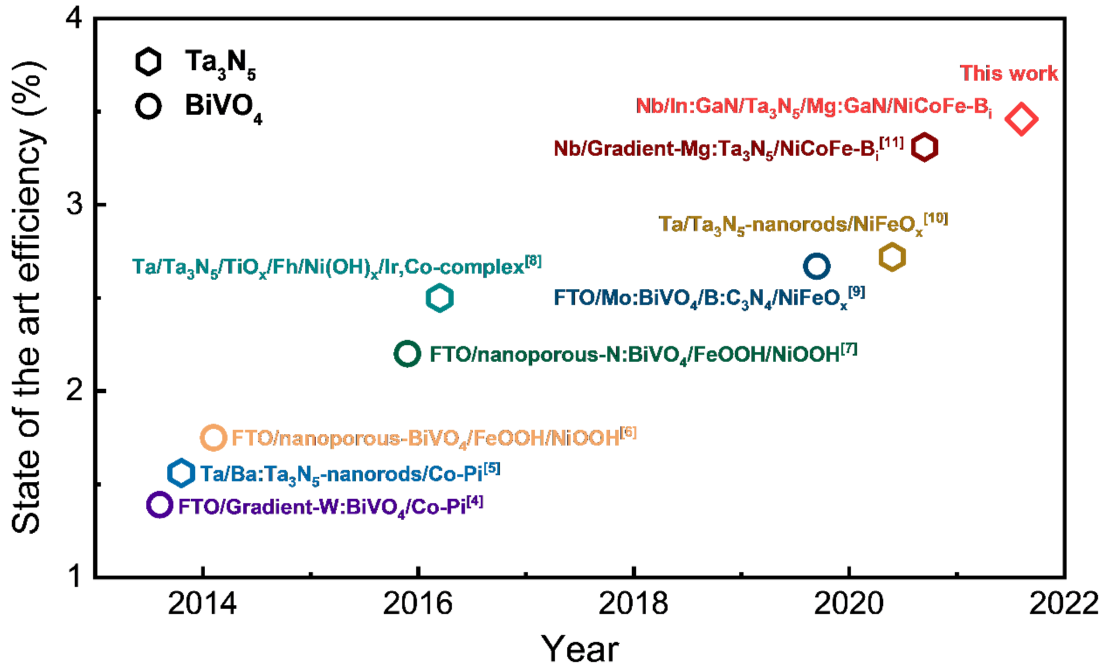

Supplementary Fig. 12 | Reported state-of-the-art ABPEs for photoanodes based on $BiVO_4$ and Ta_3N_5. The structures of the photoanodes are provided and the ABPE values are extracted from Supplementary *Ref. 4-11*.

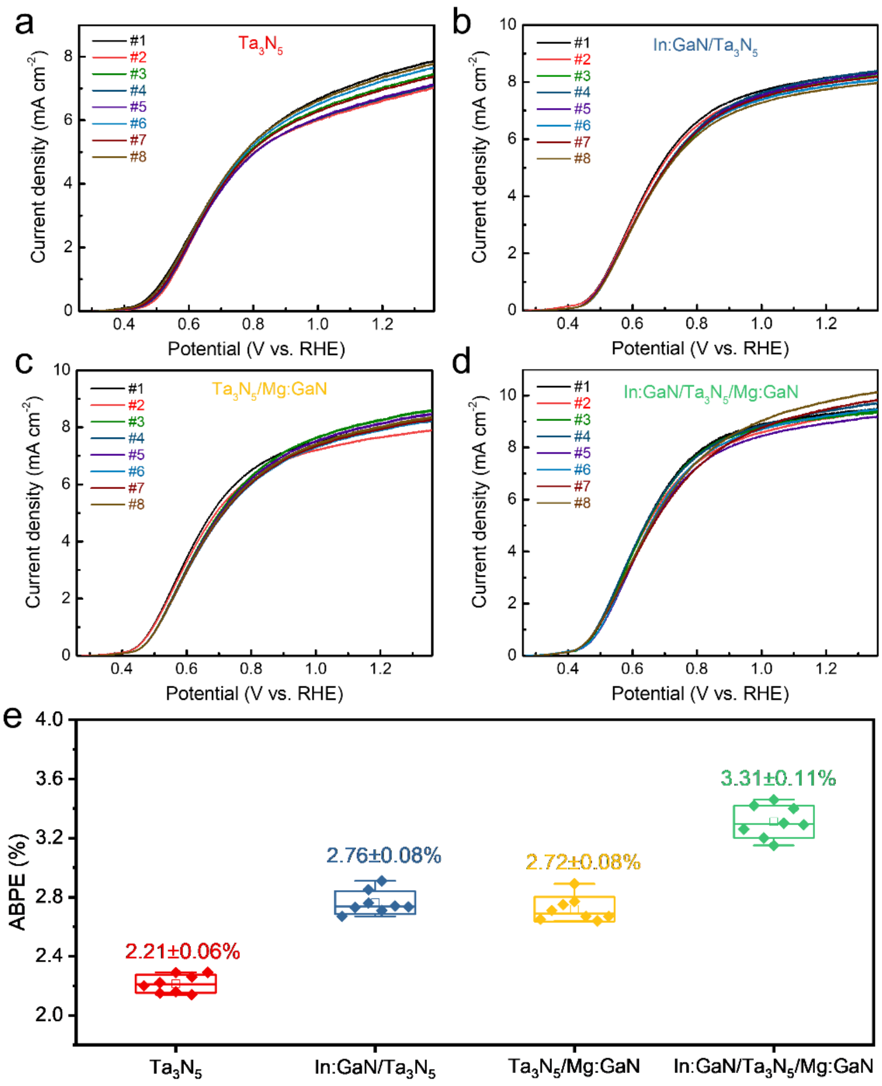

Supplementary Fig. 13 | Reproducibility of the PEC performance of Ta_3N_5-based photoanodes with different layered structures on Nb substrate. J-V curves for batches of eight photoanodes: **a,** Ta_3N_5, **b,** Ta_3N_5/Mg:GaN, **c,** In:GaN/Ta_3N_5, and **d,** In:GaN/Ta_3N_5/Mg:GaN. All the thin films were modified with the NiCoFe-B_i co-catalyst and tested in 1 M KOH electrolyte under AM 1.5G illumination. The J-V curves of the champion devices for each structure are reported in Fig. 4a. **e**, Statistics of the ABPEs of Ta_3N_5-based photoanodes with different layered structures. The average ABPEs and the standard deviations are shown in the box chart.

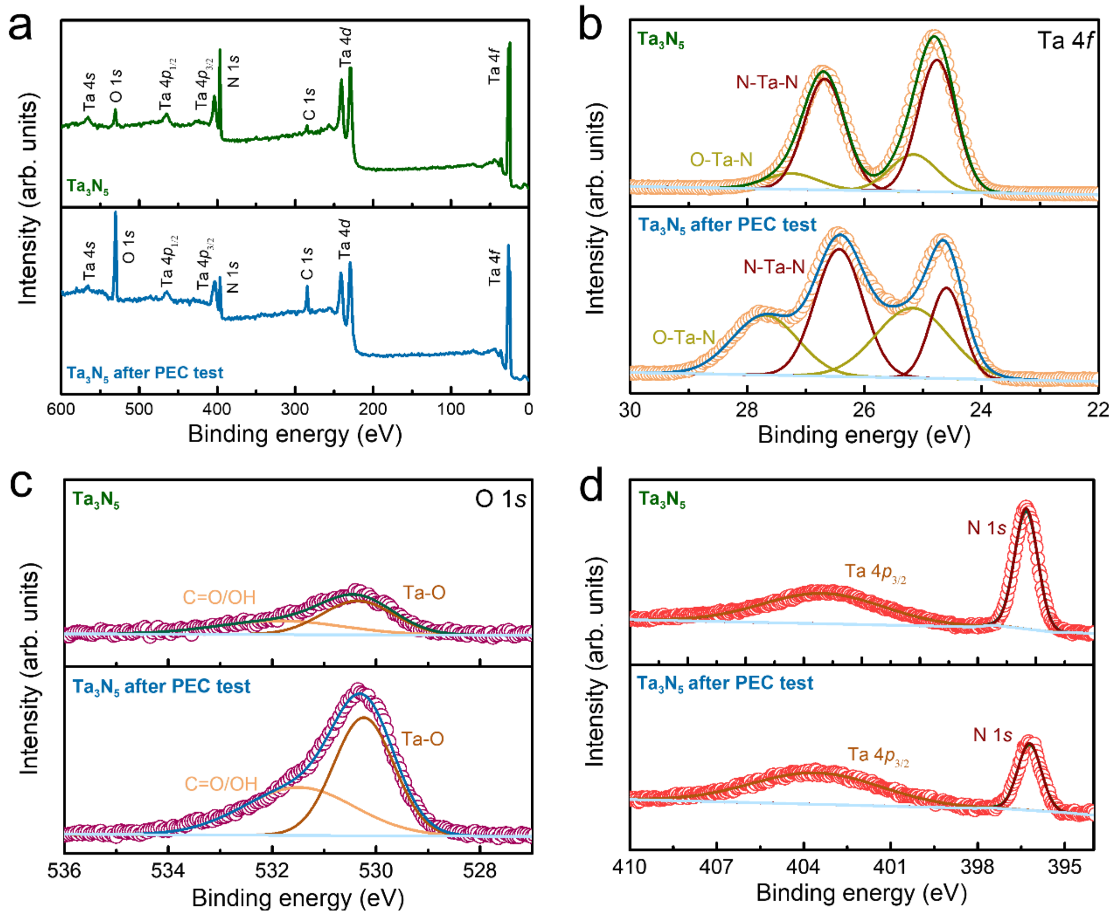

Supplementary Fig. 14 | XPS spectra of Ta_3N_5 photoanode on Nb substrate before and after PEC test. a, Survey spectra, and core-level spectra of **b,** Ta *4f*, **c,** O *1s*, and **d,** N *1s*. The Ta_3N_5 photoanode modified with NiCoFe-B_i co-catalyst was tested in 1 M KOH at 1.0 V vs. RHE under AM 1.5G for 160 min. Afterwards, the NiCoFe-B_i co-catalyst on the surface was dissolved with diluted HCl for XPS characterization. The increased O-Ta-N peaks in **b** and Ta-O peak in **c** and decreased N *1s* peak in **d** suggest the self-oxidation of the Ta_3N_5 surface, which accounts for the degradation of the photocurrent.

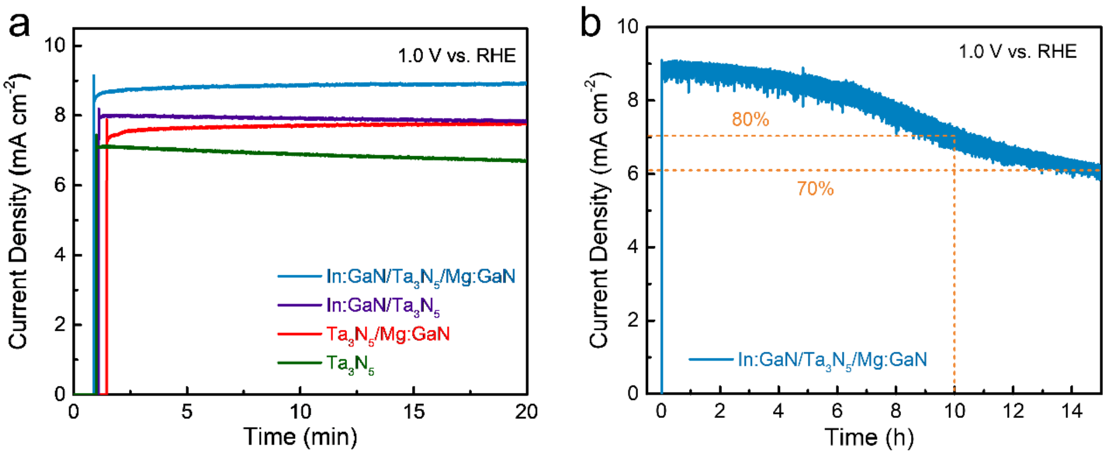

Supplementary Fig. 15 | The stability of NiCoFe-B_i modified Ta_3N_5-based photoanodes on Nb substrate. a, Steady-state photocurrent of different Ta_3N_5-based photoanodes measured at 1.0 V vs. RHE in 1 M KOH under AM 1.5G simulated sunlight. The samples with a top Mg:GaN layer (Ta_3N_5/Mg:GaN and In:GaN/Ta_3N_5/Mg:GaN) showed improved stability compared with those without a top Mg:GaN layer. **b,** Stability test of the In:GaN/Ta_3N_5/Mg:GaN photoanode at 1.0 V vs. RHE for 15 h.

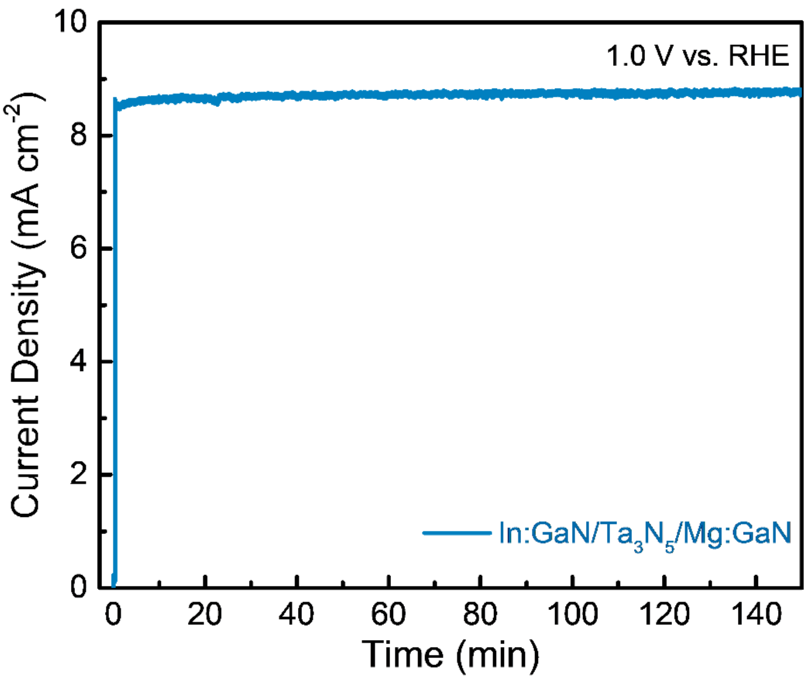

Supplementary Fig. 16 | The steady-state photocurrent of In:GaN/Ta_3N_5/Mg:GaN photoanode on Nb substrate recorded during gas chromatography measurement. The NiCoFe-B_i modified In:GaN/Ta_3N_5/Mg:GaN photoanode was held at 1.0 V vs. RHE in 1 M KOH electrolyte for 150 min.

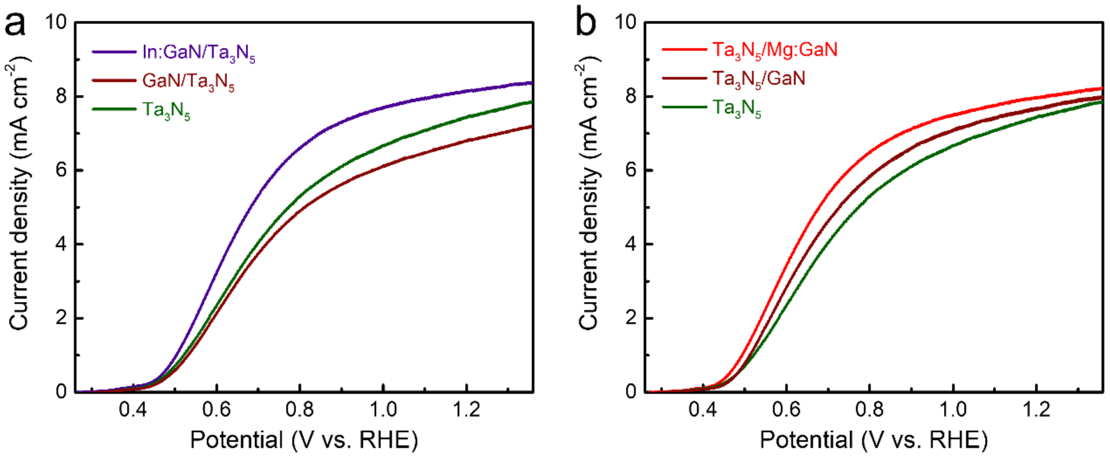

Supplementary Fig. 17 | Effect of In and Mg doping of the interfacial GaN layers on the PEC performance of the Ta_3N_5-based films on Nb substrate. a, J-V curves for Ta_3N_5, GaN/Ta_3N_5, and In:GaN/Ta_3N_5 photoanodes. **b**, J-V curves for Ta_3N_5, Ta_3N_5/GaN, and Ta_3N_5/Mg:GaN photoanodes. All the photoanodes were modified with NiCoFe-B_i cocatalyst and tested in 1 M KOH electrolyte under AM 1.5G illumination.

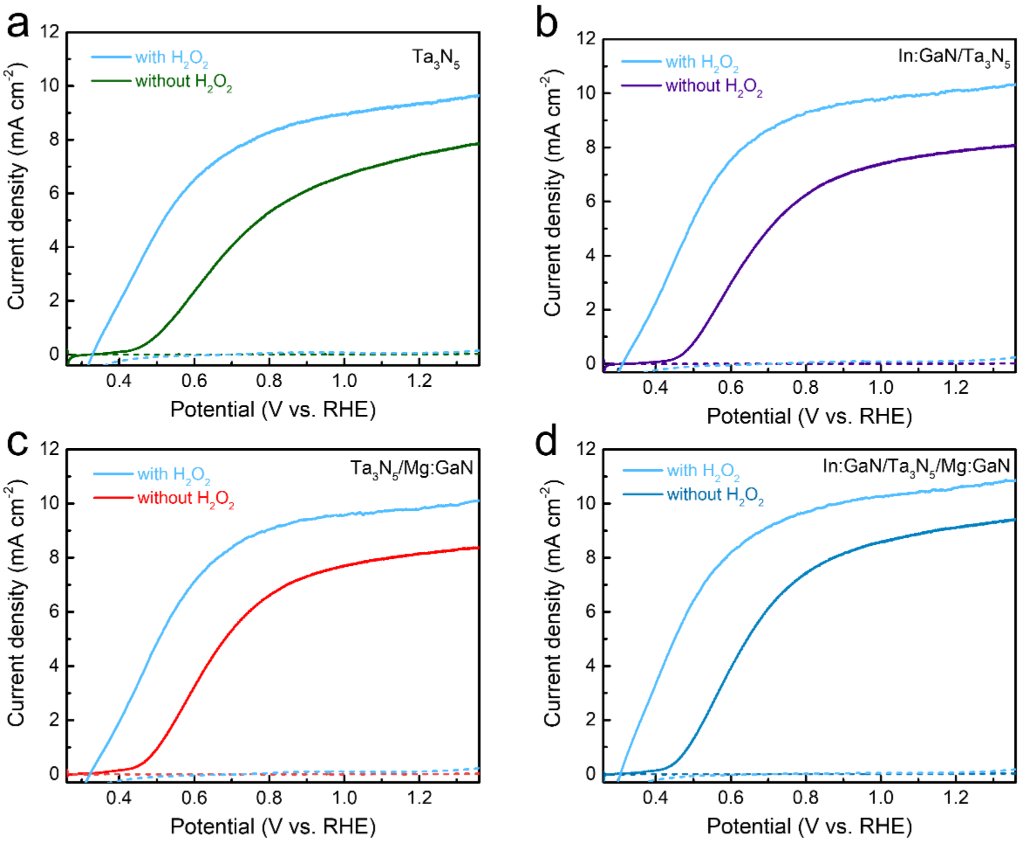

Supplementary Fig. 18 | J-V curves of different Ta_3N_5-based photoanodes on Nb substrate measured in 1 M KOH electrolyte with or without addition of 0.5 M H_2O_2. a, Ta_3N_5. **b,** In:GaN/Ta_3N_5. **c,** Ta_3N_5/Mg:GaN. **d,** In:GaN/Ta_3N_5/Mg:GaN. The samples were all modified with NiFeCo-B_i co-catalyst. The solid lines were measured under AM 1.5G illumination, while the dashed lines were measured in the dark.

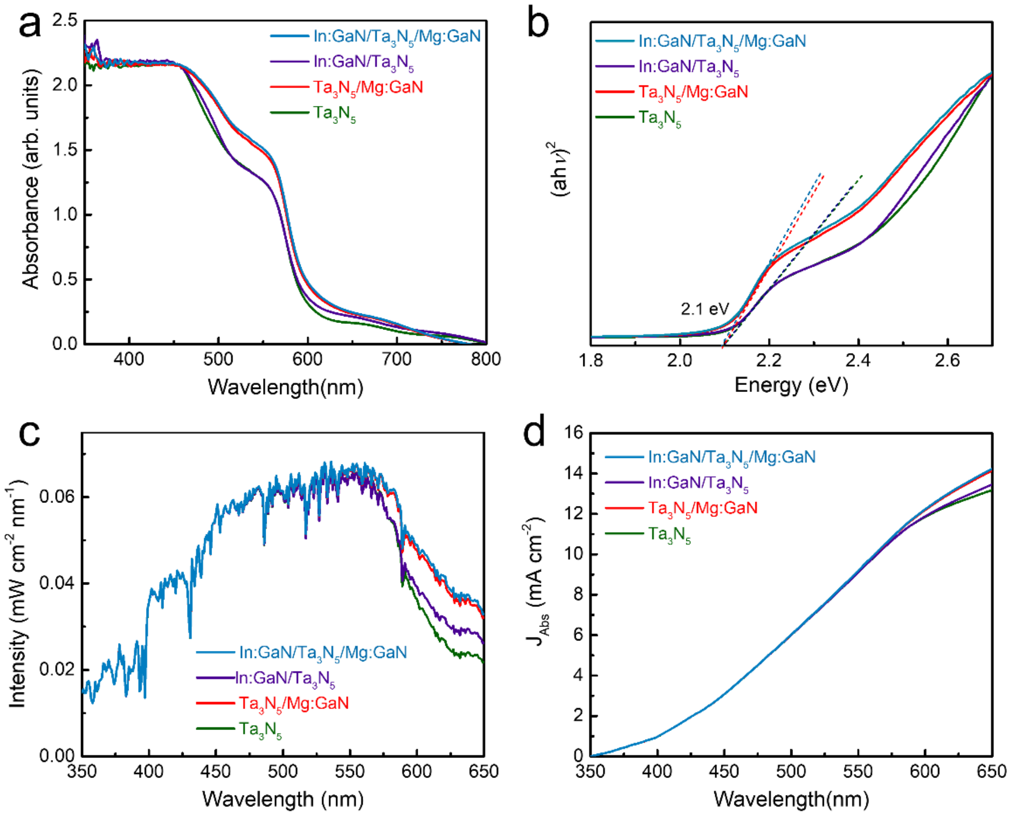

Supplementary Fig. 19 | Estimation of the maximum photocurrent density based on absorption spectra. a, UV-vis absorption spectra of different Ta_3N_5-based thin films deposited on quartz glass substrate. **b,** Tauc plots of the UV-vis absorption spectra**. c,** Absorbed photon flux spectra obtained by integrating the absorption spectra with the AM 1.5G reference spectrum**. d,** Absorption photocurrent (J_{Abs}) curves obtained by assuming 100% absorbed photon-to-current conversion efficiency.

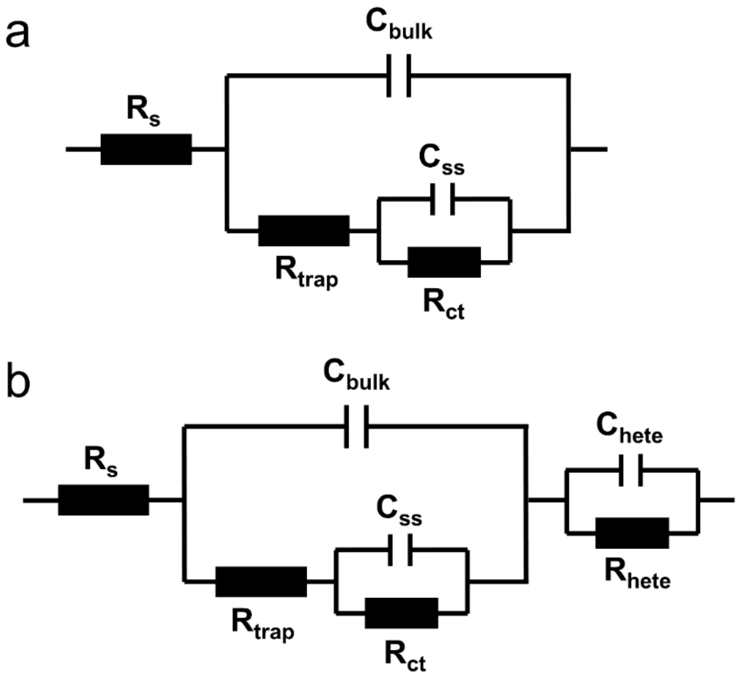

Supplementary Fig. 20 | The equivalent circuit models used for fitting the PEIS Nyquist plots. a, Two-RC-unit equivalent circuit model for fitting the PEIS Nyquist plot of Ta_3N_5 photoanode. **b,** Three-RC-unit equivalent circuit model for fitting the PEIS Nyquist plot of In:GaN/Ta_3N_5/Mg:GaN photoanode. The R_s, C_{bulk}, R_{trap}, C_{ss}, R_{ct}, R_{hete}, and C_{hete} in equivalent circuit models stand for the series resistance of photoelectrocatalysis system, the bulk capacitor of space charge region, the trapping resistance of photo-generated holes at surface states, the capacitor of surface states, the charge transfer resistance, the resistance of heterojunction structure, and the capacitor of heterojunction, respectively.

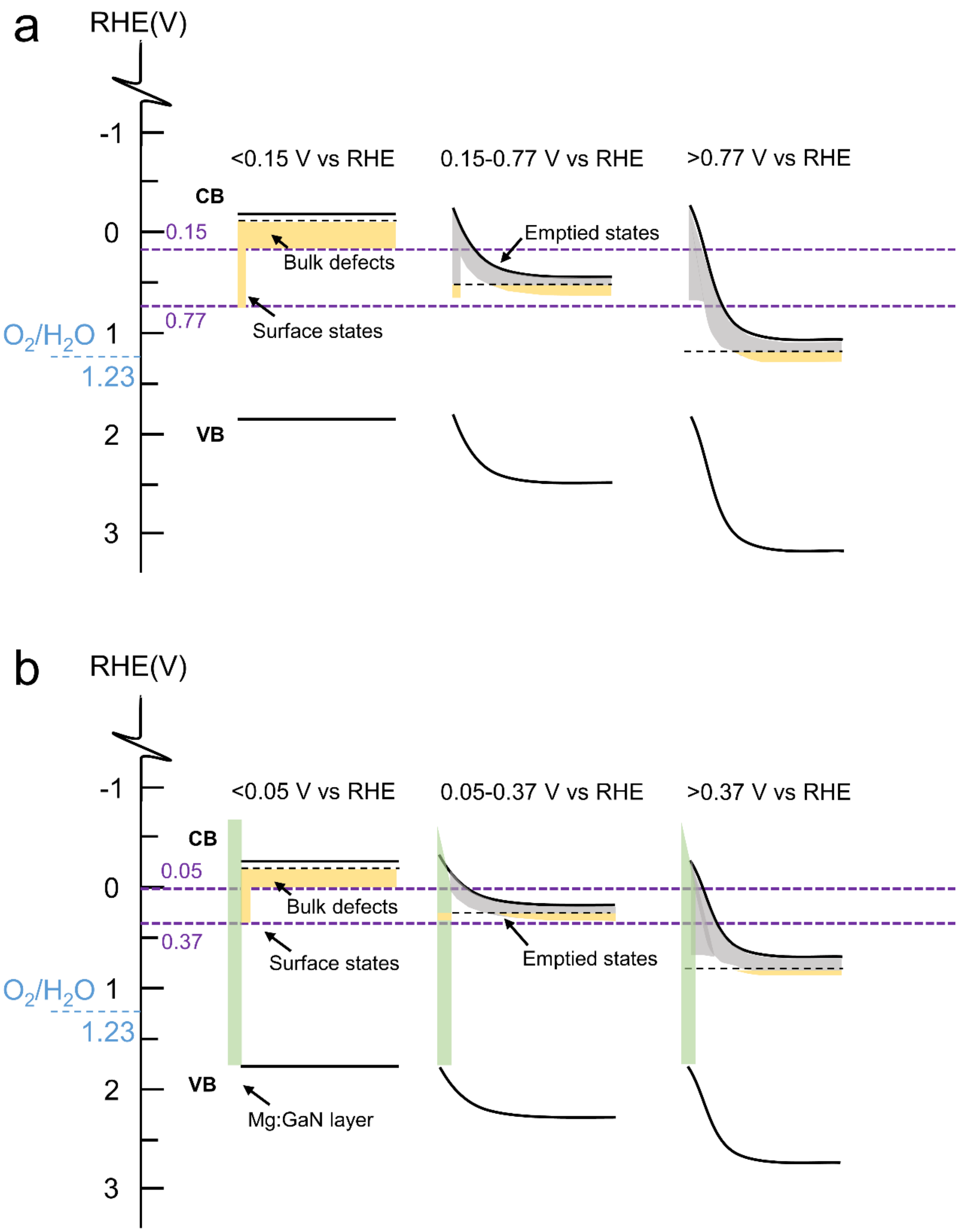

Supplementary Fig. 21 | The schematic diagrams of band bending near the photoanode/electrolyte interface under different bias potentials. a, Ta_3N_5. **b,** In:GaN/Ta_3N_5/Mg:GaN.

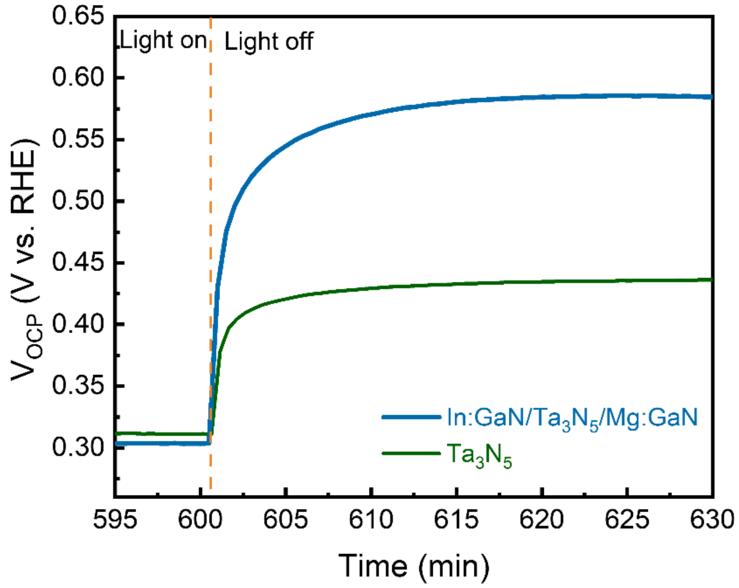

Supplementary Fig. 22 | Open circuit potential (OCP) decay profiles for Ta_3N_5 and In:GaN/Ta_3N_5/Mg:GaN photoanodes on Nb substrate under illumination and in the dark. The photoanodes were not modified with OER co-catalyst. The curves were measured in 1 M KOH electrolyte. The AM 1.5G simulated sunlight was turned off after recording the OCP for 10 min to create significant charge recombination.

Supplementary Table 1 | The detailed fitting parameters for the TRPL decay curves of different Ta_3N_5-based films.

Sample	τ (ns)	β	⟨τ⟩ (ns)
Ta_3N_5	0.206	0.370	0.862
Ta_3N_5/Mg:GaN	1.921	0.486	4.054
In:GaN/Ta_3N_5	1.752	0.477	3.837
In:GaN/Ta_3N_5/Mg:GaN	2.259	0.504	4.452

Supplementary Table 2 | The detailed parameters of the J-V curves for Ta_3N_5-based photoanodes with different layered structures.

Photoanode	Onset potential (V *vs.* RHE)	Photocurrent density at 1.23 V vs. RHE (mA cm^{-2})	ABPE (%)
Ta_3N_5	0.47	7.50	2.29
Ta_3N_5/Mg:GaN	0.40	8.03	2.89
In:GaN/Ta_3N_5	0.42	8.20	2.91
In:GaN/Ta_3N_5/Mg:GaN	0.38	9.30	3.46

Supplementary Table 3 | Fitted parameters of PEIS Nyquist plots for Ta_3N_5 and In:GaN/Ta_3N_5/Mg:GaN photoanodes.

Photoanode	R_s (Ω)	C_{bulk} (F)	R_{trap} (Ω)	C_{ss} (F)	R_{ct} (Ω)	R_{hete} (Ω)	C_{hete} (F)
Ta_3N_5	3.15	9.74×10^{-5}	449.3	3.67×10^{-4}	503.7	-	-
In:GaN/Ta_3N_5 /Mg:GaN	2.11	7.06×10^{-5}	6.0	6.66×10^{-5}	23.0	1.13	1.27×10^{-4}

Supplementary References:

1. Liu, X. *et al.* High Photovoltage inverted planar heterojunction perovskite solar cells with all-inorganic selective contact layers. *ACS Appl. Mater. Interfaces* **11**, 46894-46901 (2019).
2. DeQuilettes, D. W. *et al.* Photoluminescence lifetimes exceeding 8 μs and quantum yields exceeding 30% in hybrid perovskite thin films by ligand passivation. *ACS Energy Lett.* **1**, 438-444 (2016).
3. Lindsey, C. P. *et al.* Detailed comparison of the Williams–Watts and Cole–Davidson functions. *J. Chem. Phys.* **73**, 3348-3357 (1980).
4. Abdi, F. F. *et al.* Efficient solar water splitting by enhanced charge separation in a bismuth vanadate-silicon tandem photoelectrode. *Nat. Commun.* **4**, 2195 (2013).
5. Li, Y. *et al.* Cobalt phosphate-modified barium-doped tantalum nitride nanorod photoanode with 1.5% solar energy conversion efficiency. *Nat. Commun.* **4**, 2566 (2013).
6. Kim, T. W. *et al.* Nanoporous $BiVO_4$ photoanodes with dual-layer oxygen evolution catalysts for solar water splitting. *Science* **343**, 990-994 (2014).
7. Kim, T. W. *et al.* Simultaneous enhancements in photon absorption and charge transport of bismuth vanadate photoanodes for solar water splitting. *Nat. Commun.* **6**, 8769 (2015).
8. Liu, G. *et al.* Enabling an integrated tantalum nitride photoanode to approach the theoretical photocurrent limit for solar water splitting. *Energy Environ. Sci.* **9**, 1327-1334 (2016).
9. Ye, K.H. *et al.* Enhancing photoelectrochemical water splitting by combining work function tuning and heterojunction engineering. *Nat. Commun.* **10**, 3687 (2019).
10. Pihosh, Y. *et al.* Ta_3N_5-Nanorods enabling highly efficient water oxidation via advantageous light harvesting and charge collection. *Energy Environ. Sci.* **13**, 1519-1530 (2020).
11. Xiao, Y. *et al.* Band structure engineering and defect control of Ta_3N_5 for efficient photoelectrochemical water oxidation. *Nat. Catal.* **3**, 932-940 (2020).
